# Health information and health-seeking behaviour in Yemen: perspectives of health leaders, midwives and mothers in two rural areas of Yemen

**DOI:** 10.1186/s12884-020-03101-9

**Published:** 2020-07-14

**Authors:** Dalia Hyzam, Mingyang Zou, Michael Boah, Abeer Saeed, Chenrui Li, Shixu Pan, Jinhe Zhai, Li-Jie Wu

**Affiliations:** 1 Department of Children’s and Adolescent Health, and Maternal Health Care, Public Health College, Harbin Medical University, 157 Baojian Road, Nangang District, Harbin, 150081 China; 2 Center for Endemic Disease Control, Chinese Center for Disease Control and Prevention, Harbin Medical University, Harbin, 150081 Heilongjiang China; 3 Ghana Health Service, Private Mail Bag Bolgatanga, Upper East Region Bolgatanga, Ghana; 4 Community Medicine and Public Health, Faculty of Medicine and Health Sciences, University of Aden, Aden, Yemen

**Keywords:** Health education, Maternal health, Rural mothers, Qualitative study, Yemen

## Abstract

**Background:**

Humanitarian crises can lead to the rapid change in the health needs of women and newborns, which may give rise to a complex situation that would require various interventions as solutions. This study aimed to examine the health education and promotion patterns, health-seeking behaviour of mothers, and barriers to the use of maternal health services from public health facilities in two rural areas of Yemen.

**Methods:**

We used a qualitative approach. We conducted in-depth interviews and focus group discussions with frontline health professionals and mothers respectively. Nine in-depth interviews were conducted with the health professionals, including 4 health leaders and 5 midwives, and 2 focus group discussions with mothers aged 18–45 years in Abyan and Lahj. Thematic analysis approach was used to analyze the data in Atlas.ti (version 8) Software.

**Results:**

Our data showed that health education and promotion activities on maternal health were ad hoc and coverage was poor. Maternal health services were underutilized by women. According to the data from the focus group discussions, the poor quality of services, as indicated by inadequate numbers of female doctors, lack of medical equipment and medicines, and costs of services were barriers to use maternal health services. Moreover, the use of prenatal and postnatal care services was associated with women’s’ perceived need. However, according to the health professionals, the inadequate human resource, workload, and inadequate funding from government have contributed significantly to the perceived quality of maternal health services provided by public health facilities. Despite the identified barriers, we found that a safe motherhood voucher scheme was instituted in Lahj which facilitated the use of maternal health services by disadvantaged women by removing financial barriers associated with the use of maternal health services.

**Conclusion:**

This study identified several obstacles, which worked independently or jointly to minimize the delivery and use of health services by rural women. These included, inadequate funding, inadequate human resources, poor quality of health services, and high cost of services. These barriers need to be addressed to improve the use of reproductive health services in Yemen.

## Background

Despite the reports that maternal mortality has declined at the global level since 2000, it remains an issue of public health concern. In 2017, an estimated 295,000 maternal deaths occurred worldwide. In consequence, maternal mortality is the second leading cause of death among women of childbearing age [1]. Moreover, nearly all these preventable deaths occur in low-income and middle-income countries (LMICs). The Global Strategy for Women’s, Children’s and Adolescents’ Health (2016–2030) noted that maternal mortality can be reduced by making improvements at the provider and health system level and reducing social and structural barriers by implementing targeting interventions [2]. Besides, specific attention has been placed to address maternal and neonatal health among specialized population groups, including humanitarian and crisis settings.

Access to quality maternal and newborn health care services is a precursor for newborn and maternal survival. The evidence shows that poor maternal and newborn health status and services in conflict-affected countries is a major contributor to the burden of maternal and neonatal ill health in Asia and the Middle-East [3]. The conflict in Yemen heightened in 2010 followed by large protests, internal fighting, and war that started in 2015 and persisted to date [4]. Before the escalation of the war between the Saudi-led government coalition and Houthi rebels, Yemen was already the poorest country in the Middle East and one of the world’s most impoverished. The humanitarian crisis has negatively affected every important sector in Yemen, including health. Between 2013 and 2016, the war and siege had a devastating impact on several maternal and child health indicators in Yemen, including a decrease in vaccine coverage for children 12–23 months, increase in the incidence of diarrhoea, anaemia and mortality among children under 5 years of age, and a rise in maternal mortality [4]. The delivery of routine primary healthcare services by the already fragile health system has also been dwarfed by the urgency of responding to the food insecurity threat and recent cholera epidemic, leaving pregnant women and newborns with limited access to a broader range of maternal and newborn health services [4, 5]. Indeed, Yemen’s health system is dominated by the public health sector but only about half of public health facilities in Yemen are fully functional, and even these face severe shortages in both medical and non-medical consumables, placing lives at risk [6]. More important is that the delivery of reproductive health care services in Yemen is limited by significant inequalities in availability and access between rural and urban women. There are reports that there is poor coverage of health care facilities, specialized staff, essential medical supplies, and funding on health, in the rural areas of Yemen where about 70% of the total population live. Consequently, only 35% of women in rural areas of Yemen, had access to skilled health professionals for maternal health care services [7].

The use of maternal and newborn health services is a complex phenomenon, which is not only influenced by the availability of, and access to primary healthcare services, but also the community and individual-level factors, including socioeconomic status, place of residence, and knowledge or awareness on the significance of maternal health services [8–12]. In Yemen, findings suggest that nearly half or more of women deliver at home and only one in five women receive postnatal care check-up after delivery [13, 14]. Inadequate awareness is obvious, which is compounded by a deteriorated healthcare system. This is because, in one of the studies, the main reason mentioned by respondents for delivering at home was because the women considered home a better place for delivery compared with the health facility and their first reason for not having postnatal care check-up was the absence of complications requiring professional care [13]. A prominent individual-level factor widely described by the literature to positively influence maternal health services utilization is high maternal education [8, 9, 15]. The premise is that comparatively, highly educated women are more knowledgeable about health problems, know more about the availability of healthcare services, have better access to health services, have more freedom to make health-related decisions, and use health information more effectively to achieve or maintain good health status.

At the same time, empirical evidence says that exposure to information on maternal health increases women’s knowledge and understanding of maternal health. Therefore, health promotion activities that promote the use of maternal and newborn services are key determinants for acceptability and use, especially in rural areas which are usually characterized by poor and less educated women [16, 17]. In other words, there is substantial literature which shows that women in LMICs who had good knowledge on health-related issues, including causes of maternal deaths, the danger signs of pregnancy and among newborns sought healthcare early and more frequently compared with women who had poor knowledge about maternal and newborn health [18–21]. Notwithstanding, qualitative findings have hinted that health education at public health facilities in urban Yemen where women receive maternal and child health services was either lacking or ineffective on account of frequency, method of delivery, the visual materials used, and targeted topics [13]. On the demand side, delay in seeking medical treatment is a common problem in Yemen and other LMICs, which is more broadly related to poor knowledge about signs and symptoms of diseases, and perception about disease severity [22–24].

The interest in investigating health-seeking behaviour has been necessitated by the increase in the coverage of healthcare services globally and widespread access to health information from traditional sources, which are often unreliable and misleading. Information can be gained passively. Thus, women do not have to necessarily actively seek information to have adequate information on maternal health. However, the nature and accuracy of the gathered health-related information could change the knowledge, belief and attitude of the information seeker regarding specific health-related behaviours [25]. The effect of the ongoing war in Yemen on health education and health information pattern of health providers, as well as the health-seeking behaviour of mothers in Yemen during pregnancy and delivery, has not been assessed. Also, by understanding the barriers affecting information dissemination and health-seeking behaviour, strategies that would address these barriers can be implemented, which can lead to an improvement in the health of the patients.

This study sought to document the experiences of health leaders, midwives, and mothers in Abyan and Lahj governorates of Yemen, relating to the health information provided around pregnancy and women’s health-seeking behaviour. We also examined the facilitators and barriers to the use of maternal health services by rural women. The findings would be important for planning purposes by the Ministry of Health and Population (MoPHP) of Yemen to improve maternal health care utilization among rural mothers in Lahj and Abyan governorates.

## Methods

### Setting

Yemen is divided into 22 governorates namely Abyan, Aden, Al-Baidha, Aldhalae, Al-Hodeida, Al-Jawf, Al-Mahrah, Al-Mahwit, Amran, Dhamar, Hadramout, Hajjah, Ibb, Lahj, Mareb, Reimah, Sadah, Sana’a, Amanat Al Asimah, Shabwah, Socotra, and Taiz. Each governorate has its own local health department which reports to the central Ministry of Public Health and Population (MoPHP). According to the most recent Yemen Demographic and Health Survey (DHS), 70% of the population of Yemen lives in rural areas [26]. We purposively selected Abyan and Lahj governorates for this study. These two governorates are among few other governorates with relatively low coverages of maternal health indicators. Moreover, they were comparatively safer for the research amidst the ongoing conflict.

### Study design and sample selection

We chose a qualitative study design for this study because it has the ability to explore participants’ perceptions and attitude, through a prepared discussion guide, which covered the areas of interest. This approach is able to explore the research question under investigation by answering questions of “what”, “how”, and “why” from participants’ perspectives [27]. We structured the issues which emerged from the respondents’ narratives into subcategories.

In the first instance, two health leaders from each of the health offices in Abyan and Lahj were purposefully identified and selected for the interviews. Then, the names of midwives of maternity and childhood health service centres in Abyan and Lahj, and the general hospital in Lahj, who worked directly with pregnant and postpartum women, were requested and invited to participate. The general hospital in Lahj was included to ensure a representativeness of health facilities in our study.

The selection of mothers was guided by their experiences with the use of ANC and delivery care services from public health facilities. We collaborated with health volunteers in two purposively selected reproductive health facilities, who helped with the recruitment of mothers for the FGDs. A quota of 10 participants was discretionally allocated to each maternity and childhood health centre. The volunteers were informed of the study aims and the characteristics of mothers required for the FGDs. The mothers who presented to the each maternity and childhood health centre were invited to participate in the FGDs on a later date. Of the 20 participants who were approached to participate, 15 accepted to take part in the present study. Altogether, 24 individuals participated in the present study, including 4 health leaders, 5 midwives, and 15 mothers aged 18–45 years (Table 1).

**Table 1:** Number and type of respondent by study site

Method	Type of respondent	Abyan	Lahj	Total
In-depth individual interviews (*n* = 9)	Health leaders	2	2	4
	Midwives	2	3	5
Focus Group Discussion (*n* = 2)	Mothers	8	7	15

### Data collection methods and tools

Two IDI guides and one FGD guide were used to collect data from the respondents (See supplementary material 1). These guides included questions and probes related to the aims of the study but also allowed for flexibility. The IDI guides for health leaders and midwives covered similar sets of topics to examine the existing health education and promotion activities and challenges faced in disseminating maternal health information in rural areas. The FGD guide explored mothers’ perceptions on maternal health and their health-seeking behaviour around pregnancy and delivery. The guide was also used to confirm information on health education and promotion given at the public health facilities.

The interview guides were designed by the authors in the English language. The first and fourth authors are fluent in written and spoken Arabic. Therefore, the approved guides were translated into Arabic by the first and fourth authors. These guides were piloted among several respondents, who were similar in characteristics to the participants in the final study. Ambiguous questions were revised. The first and fourth authors conducted all the interviews and FGDs in Arabic. Written and verbal consent were obtained from the health care professionals and women, respectively before interview. The purpose of the study, including the benefits and risks were communicated to the participants. In addition, participants were informed about their right to withdraw from participating in the study. All the interviews were tape-recorded with the permission of participants. Notes were also taken during the interviews. To ensure anonymity, respondents’ names were replaced with pseudonyms. The names of the institutions where the health leaders and midwives worked were also excluded. The interviews were carried out between August 2018 and October 2018. The venues for the interviews were determined by the respondents. In total, nine IDIs were conducted with health providers and two FGDs with mothers of the reproductive age (15–49 years).

### Data analysis

All interviews were audio- recorded, transcribed verbatim in Word™ and uploaded into Atlas.ti (version 8) Qualitative Data Analysis (QDA) Software for the analysis. An inductive and deductive thematic content analysis was applied to the data. The analysis was done by first, reading the transcripts repeatedly to understand the data, identify overall themes as well as summarizing the responses to each vignette. Open coding was conducted by highlighting pieces of the text that were of interest to the study objective and the emerging themes and sub-themes were discussed within the research team. This was followed by axial coding to establish similarities between themes. We ensured that there was enough data to support each theme. As result, some themes were collapsed because they were unifying, renamed or even deleted. We paraphrased responses from respondents and applied quotations where necessary.

### Validity and reliability of the data collected

Threats to validity and reliability of the information collected were addressed by collecting rich data. The data collectors (first and fourth authors) are experienced with the collection of qualitative data using IDIs and FGDs. The data collection tools were assessed and modified by experts in the Department of Children’s and Adolescent Health, and Maternal Health Care, Harbin Medical University. The data collection tools were pre- tested and modified before actual data collection. All the interviews were audio-recorded and transcribed verbatim. Detailed field notes were taken during the interviews and feedback was solicited from participants by sharing the detailed notes with the participants for a consensus on the interpretation of participants’ opinions. We triangulated the data sources and data collection tools to ensure the validity and credibility of the information collected from the respondents; the IDIs with health leaders and midwives provided information on the health education process and delivery of maternal health services from the provider’s perceptive; the FGDs with mothers highlighted women’s own experiences with the utilization of health care services and barriers to access, as well as the health information given around the period of ANC and delivery.

### Ethical considerations

This study was approved by the Research and Ethics Committee of public health college, Harbin Medical University, Harbin, China. Also, the approval letter was given by the Ministry of Health and Population of Yemen (2018/100667–8) to conduct this study. Written informed consent was obtained from the health leaders and midwives. However, the mothers gave their consent orally. Permission was obtained from respondents before recording the interviews. The confidentiality and anonymity of the participants were ensured.

## Results

### Profile of the respondents

The majority of mothers attained less than secondary level of education (8/15) and were unemployed (12/15). By contrast, the majority of midwives (3/5) and all the health leaders (3/3) had at least secondary level of education. The number of years of professional experience for the midwives ranged from 2 to 32 years and the majority (4/5) had served for more than 5 years (Table 2).

**Table 2:** Characteristics of participants in in-depth interviews and focus group discussion

Characteristic	Mothers (*n* = 15)	Midwives (*n* = 5)	Health leaders (*n* = 4)
Age, mean (SD)	29.2 (6.8)	50.8 (8.2)	47 (8.8)
Highest level of education, n (%)			
No formal education	4 (27)	0 (0)	0 (0)
Basic education	4 (27)	2 (40)	0 (0)
Secondary education	5 (33)	2 (40)	1 (25)
Tertiary education	2 (13)	1 (20)	3 (75)
Employment status, n (%)			
Employed	3 (20)	5 (100)	4 (100)
Unemployed	12 (80)	0 (0)	0 (0)
Number of children, n (%)			
1	6 (40)		
2	5 (33)		
3–4	4 (27)		
Number of years of professional experience, n (%)			
≤ 5 years		1 (20)	2 (50)
> 5 years		4 (80)	2 (50)

*SD* Standard deviation

### Emerging themes

The findings presented are based on individual in-depth interviews with 9 health professionals and 15 mothers.

### Ad hoc and/ or parallel health education and promotion activities

In general, health promotion activities at public health facilities were integrated into routine health programming. However, the leaders reported that national capacity to plan and implement comprehensive Social and Behaviour Change Communication (SBCC) strategies and general health promotion interventions was low. They expressed concern about the sustainability of international organizations-led health promotion programmes in some target areas, which are emergency-related and the general poor integration of health promotion in routine health programming. A health leader explained:

*“After the war] in 2015] the health information process is drawn by the international health organizations which have focused on remote areas and some regions. Health education programs are not planned. Most of the health education programs are now activated only during emergencies, such as the recent epidemic of cholera and diphtheria”* (Health Leader 2; Abyan).

Some trained frontline health workers reported that the ongoing war had impacted negatively on delivering health education through mass media approaches. Instead, health education through interpersonal means with clients was now being used occasionally by health volunteers. A Midwife explained as follows:

*“Before this civil war, the situation in this hospital ]General hospital/Lahj[ was better. We] midwives[ had given health messages to mothers through TV shows. Now, we sometimes have to collect mothers downstairs ]waiting hall[ to give them health education. This is done randomly by health volunteers”* (Midwife 2; Lahj).

### Poor coverage of health promotion programmes

Health Leaders mentioned some challenges to the provision of adequate health care and health promotion messages to clients, including inadequate human staff and related workload, inadequate funding for health and health promotion interventions, poor planning and vertical programme implementation of health facility-based and mass campaigns (especially for maternal health programmes). Some health leaders explained:

*“The inadequate numbers of human resources, workload, and poor financial and motivational support among health providers are some of the challenges limiting our ability to provide adequate health care services and sufficient health education to mothers”*(Health Leader 2; Lahj).

*“The management and planning regarding maternal health education process are poor. Additionally, the health information that is provided during health campaigns and at the reproductive healthcare centres aren’t integrated”* (Health Leader 1; Lahj).

Health leaders suggested the collaboration between stakeholders in Primary Health Care and Health Promotion in the review, planning and implementation of health campaigns (through facility-based and mass campaign approaches) as a means of addressing the challenges of health promotion and education in Yemen.

*“Unifying the efforts between the health stakeholders of the primary health care and health education programs could identify the health education implementation problems and that might improve the way of health messages transferring process”* (Health Leader 1; Lahj).

Women’s health-seeking behaviour: facilitators and barriers to the use of maternal health services.

The discussions with the health professionals and mothers identified many factors, which were perceived as facilitators and barriers to the use of maternal health services by rural women in the study area. Although these factors interacted with each other, we classified them into three main categories, including policy or system factors, provider-related factors, and patient-related factors.

### Policy or system-related factors

i) Inadequate funding from government

The narratives of the health professionals showed that the public health facilities received inadequate funding from the government for the provision of basic and essential services.

*“The government has not been able to support health care services. The funding for health activities is woefully inadequate. Around 25% of the financial support comes from the government and 75% comes from the Non-governmental organizations and the international health organizations’. Because of the inadequate funding, the health facilities are not able to procure essential logistics and medicines to provide care”* (Health Leader 2; Lahj).

ii) Health financing scheme: Safe motherhood voucher scheme

The data showed that there was a health financing scheme—safe motherhood voucher— which enabled access to services by reducing financial barriers for women and their families while providing financial support to both private and public health facilities to improve the quality of services provided. Furthermore, it was noted that health providers were more responsive to women covered under this scheme. However, it was also revealed that the scheme was limited to only poor rural women. Therefore, not all the women and their families benefited from the scheme. Besides, not all the health facilities were contracted under this scheme.

*“Because of the Safe motherhood voucher scheme, women are now able to access health services without any financial difficulties. Apart from that, we****]****midwives****[****visit the mothers who have been included in the voucher scheme on the third day after childbirth to make sure that there is no puerperal fever and on day 30 to provide family planning services. To me, this scheme has enabled rural women to access reproductive health services, even during the conflict. The only limitation is that the scheme does not include all women”* (Midwife 1, Lahj).

### A mother confirmed part of the narrative of the midwife

*“When I delivered and returned home from the health facility, the midwives visited me two times at home. In the first visit, they checked the baby’s health. Then they came again and talked to me about family planning methods. They [midwives] also told me to visit the health centre if I faced any problems”* (Mother 3, 30-34 years old, Lahj).

### Provider-related factors

i) Inadequate human resource and medical equipment

Our analysis showed that the public health facilities in the study area did not have an adequate human resource and essential equipment to provide the needed maternal health services to women during pregnancy. As a result, some women delayed first antenatal care attendance until after 3 months gestation.

*“There are many challenges that are preventing us [health providers] from providing basic and essential health services to pregnant women. We don’t have adequate female doctors and midwives, drugs, and essential equipment to provide services. For instance, this whole department [CEmOC-General hospital] has only 12 formal midwives, 10 formal female doctors, and around 10-12 health volunteers. This is inadequate and most of the time we are faced with increased workload”* (Midwife 2; Lahj).

One mother lamented:

*“During my first pregnancy, I was going to the health centre in the first three months. But most of the time, I did not meet the doctor. So in my second pregnancy, I did not visit early again because I was not having any problems. I did not want to go and not meet the doctor again. Even I did not do physical examination when I went there”* (Mother 3; above 45years old; Abyan).

It was further noted that although some pregnant women decided on their own to delay first antenatal care, others were advised by the health providers to delay their first attendance due to the unavailability of doctors. A mother had this to say:

*“The doctor is not always available, because of that the midwives told us [pregnant women[ that we should attend the maternity and childhood centre when the pregnancy is about four months old. So that is what we [pregnant women] also do* (Mother 2; 25–29 years old; Abyan)

ii) Cost of services

The cost of health services was a perceived barrier to the use of public health facilities by some women as revealed in the interviews with the health professionals. It came out that there were high financial costs associated with obtaining antenatal and vaccination cards, as well as receiving some services, such as physical examinations, which prevented women from frequently using the reproductive health facilities.

*“The cost of some health care services in the facilities prevent mothers from visiting the reproductive healthcare centres frequently. Mothers have to pay about 3000 Yemeni riyals [about $6] to obtain antenatal care, and vaccination cards and to receive health services like physical examinations. This to me is too much for them because most of these mothers cannot afford this amount. After the war in 2015, folic acid was not available in the public health facilities for a while and the ministry of health did not also supply the tetanus vaccine, which we give to mothers”* (Health leader 2; Lahj).

### Patient-related factors

i) Perception of public health facilities and quality of care

The discussions showed that women’s perception of public health facilities and quality of care influenced their health-seeking behaviour during pregnancy. Generally, public health facilities were perceived to provide low-quality services due to inadequate critical staff, equipment, and essential medicines. In addition, although private health facilities were preferred over public health facilities, their use was associated with being able to afford the services.

*“In the first three months of pregnancy, the midwives always check the health status of the woman and the baby but because of the lack of female doctors, it is better to go to the private clinic. If I don’t have enough money to go to the private clinic I go to the maternity and childhood centres to check the health of my baby, I don’t have any other option”* (Mother 4; 25-29 years old; Abyan).

*“If I know I am pregnant I do the physical examination in the private clinic until I feel that I am good then I go to the reproductive healthcare centres in the second trimester”* (Mother 1; 25-29 years old; Lahj).

Women also alternated between public health facilities and private facilities to receive care.

*“In the first month of my pregnancy, I went to the private clinic to do the blood test and after two months, I went to the maternity and childhood health centre for ‘echo’****]****Fetal echocardiography****[****. Then I returned to the reproductive healthcare centre after two months to continue receiving the other services”* (Mother 2; 25–29years old; Abyan)

The interviews with the midwives confirmed the narratives of the mothers.

*“We don’t have enough female doctors. In addition to that, the services we provide are below standards, they are poor in terms of quality. Because of that the mothers who are highly educated and can afford services, prefer to visit the private health facilities to receive reproductive health services”* (Midwife 3; Lahj)

ii) Early antenatal care is associated with a perceived need

According to the data, there was a consensus on early ANC attendance in the first trimester of pregnancy. Nonetheless, women’s use of ANC in the first trimester in public health facilities was associated with women’s perceived need for services, which was shaped by the presence of obstetric complications, availability of female doctors, and medical interventions during pregnancy, such as folic acid supplementation.

*“It is necessary to go to the health centres from the first month of pregnancy for the midwives to check the pregnancy. I went to the maternal health centre in the first month of my recent pregnancy to get the folic acid but was not always available. When I was pregnant, I visited the health centre about 3-4 times before I delivered”* (Mother 7; 35-39 years old; Lahj).

*“If I don’t have any complications, I don’t think I will go to the reproductive healthcare centre. I started visiting the reproductive healthcare centre in the fourth month of my pregnancy because there are no female doctors and the centres do not have some of the equipment to perform some of the tests”* (Mother 3; above 40years old; Abyan).

One of the midwives explained why all the essential services were not provided to pregnant women in the first trimester of pregnancy:

*“One of the reasons why we are not able to provide all the health services to pregnant women in the first trimester of pregnancy is because of the shortage of female doctors. But sometimes we provide health counselling and folic acid to some of the pregnant when they visit the health facility within the first trimester of pregnancy”* (Midwife 1; Abyan)

The health providers also revealed that there were misconceptions about the use of folic acid during the pregnancy. The results showed that mothers perceived that taking folic acid during pregnancy increased the size of the brain of the fetus. Therefore, some mothers declined to take folic acid during pregnancy. This they said, was more common among mothers with inadequate education.

*“Mothers who are more educated have good knowledge about the medicines we provide during pregnancy. There is a misconception among mothers on the benefit of folic acid during pregnancy. They [mothers****[****think that the folic acid increases the brain size of the fetus, that’s why some of them refuse to take the folic acid during pregnancy. This is very common among the mothers who are not educated”* (Midwife 2; Abyan).

iii) Postnatal care was not necessary without complications

The majority of mothers (13/15) perceived that PNC check-up after delivery was associated with complications. For that matter, there was no need for PNC services if there were no complications with the mother or newborn after delivery. The data also showed that mothers visited the health facilities to receive vaccinations, contraceptive, or seek care for a sick newborn. One of the participants responded:

*“Visiting the health centre after childbirth is not important if there are no complications”* (Mother 4; 25-29 years old; Abyan).

Nonetheless, one mother opined that it was important for mothers to receive PNC even when complications did not set it

“*I went to the health centres after delivery to check-up my health status. I did not have any complications after childbirth, but I think mothers should always go to the health centre to check-up their health status after delivery”* (Mother 6; 30 -34years old; Abyan).

However, the discussions with the midwives demonstrated that PNC was also not provided at the health facilities for mothers after delivery. Further, midwives invited mothers whose pregnancies were somewhat marked with complications to attend the health facilities after delivery for a checkup

*“In general, there is no physical examination for mothers after delivery at the reproductive health care centres. We [midwives] just provide blood sugar test for mothers who need sub-dermal contraceptive. Mothers come after delivery if they feel tired, her baby is sick, for vaccination, and family planning services”* (Midwife 3; Lahj).

*“I ask the mothers who had some health problems during the time of pregnancy to visit the health centres after childbirth to check their [mother] health status’.* (Midwife 1; Abyan).

## Discussion

This study aimed to examine the health education and promotion activities, health-seeking behaviour of mothers, as well as facilitators and barriers to the use of maternal health services from public health facilities in two Abyan and Lahj governorate of Yemen. Overall, our analysis showed many factors, including factors relating to policy, health providers, and patients, act independently or jointly to facilitate or inhibit the delivery and use of maternal health services in the study area. However, more generally, the onset of fighting in Yemen has led to the deterioration of the health care system. There have been both direct and indirect attacks on health facilities, which are depended on for service delivery [28]. Besides, staff inadequacies, particularly the lack of female doctors in most public health facilities, and a disproportional distribution and underutilization of the available health staff, and lack of medical consumables and non-medical consumables, have been reported from an assessment of public sector emergency obstetric and neonatal care in Yemen [7].

The receipt of maternal health information has been associated with the use of maternal health services [29]. Our study showed that maternal health education and promotion activities were integrated into routine health programming. However, the provision of adequate health information to women was limited by inadequate human resource, workload, and inadequate funding. Consequently, maternal health education activities were ad hoc and sometimes relied on volunteers to educate women on reproductive health matters. In addition, mass media approaches, which were previously used for health education and promotion, were no longer utilized due to the impact of the conflict, thus resulting in poor coverage of maternal health information. There were also concerns about the sustainability of the health promotion programmes developed by international organizations, which only targeted certain rural areas of Yemen. The use of health volunteers in health promotion interventions has shown some effectiveness [30]. It has the advantage of allowing for a two-way interaction compared with mass media, which is one-way communication. However, the concern lies in the depth and accuracy of the information being passed on by the volunteers to the clients. Reports have recognized that volunteers performed less effectively in more complex tasks such as counselling and require regular supervision, training, and adequate resources to perform successfully within the health system [31].

The results showed that both supply and demand factors play an important role in the health-seeking behaviour of women. The acceptability of using a health provider is affected by women’s perceptions of the provider [32, 33]. The experience of poor quality services—particularly when private health providers are perceived to provide better services—often resulted in the suboptimal utilization of prenatal and delivery care services from public health institutions. We found that rural Yemeni women’s use of the public health system was limited by their perceived poor quality of services, as indicated by the lack of critical staff, particularly female doctors, equipment, and essential medicines. Our analysis further showed that women in the study area preferred private health facilities over public health providers, although the actual use of private care was highly determined by the purchasing power of the women. Similar observations were noted among women in Ghana [34]. More broadly, the private health sector appears to flourish everywhere, which may be attributed to its comparative advantages, such as easier access; shorter waiting time; flexible opening hours; better availability of the human resource, drugs, and medical equipment; and better staff attitude [35, 36]. Despite this, there is evidence from other developing countries that suggests that the care provided by private health entities did not differ significantly from that provided by public health entities, in terms of quality [37, 38]. Therefore, we strongly believe that the situation in Yemen may have been exacerbated by the disproportional distribution of health staff between rural and urban areas and inadequate and erratic financial support to the public health system by the government [7, 28]. Data from The World Bank showed that before the onset of fighting in Yemen in 2015, the government’s health expenditure as a percentage of the Gross Domestic Product (GDP) had decreased from approximately 6% in 2009 to 4.2% in 2015 [39].

In this study, the cost of services was mentioned by health providers and mothers as a perceived barrier to the use of maternal health services in both private and public health facilities. Cost of services has undoubtedly been a major barrier to the use of health services among rural women in other low and middle-income countries worldwide [10, 13, 32]. We found that the expenditure on ANC and vaccination cards, as well as physical examinations during pregnancy, turned out to be cumbersome for rural women in Yemen. Women’s use of private health facilities as an alternative was also limited by cost. However, we identified that a voucher scheme, which was similar to an insurance scheme in many ways, was instituted by the Yamaan Foundation for Health and Social Development in Yemen, with support from Options Consultancy Services. This health financing scheme aimed to reduce the financial barriers that women faced in accessing reproductive health services while channeling funds to expand the capacity of health facilities to provide safe motherhood services. According to the reports, the voucher programme improved the use of maternal health services by enabling women to access care from other health providers, including privately owned health facilities from nearby districts during the worse days of fighting, when several public health facilities were forced to close down [40]. Unfortunately, this scheme was limited to only disadvantaged women in Lahj and was not available to women in Abyan. A similar voucher scheme has been associated with a significant improvement in the use of delivery services by low-income women in neighbouring Pakistan [41].

Prenatal care from a skilled provider is important for early detection of disorders to reduce risks for both mother and baby during pregnancy, at delivery, and during the postpartum period. Moreover, both direct and intermediate effects of ANC on the subsequent use of health facilities for delivery and postpartum care services have been reported [33]. For various reasons, the WHO recommends early ANC for all women from low and middle-income countries [42]. However, our analysis showed that women’s use of public health facilities for ANC and PNC was associated with perceived morbidity. We noted that women associated early seeking of care during pregnancy with experience of complications during ongoing pregnancy, which was broadly aligned with findings from a study in Sana’a, Yemen [43]. On the other hand, postnatal care check-up was limited by the lack of services at the health facility and mothers’ perceived need for PNC. Obstetrical need, as indicated by complications during an ongoing pregnancy and or negative outcome of previous pregnancies has been associated with women’s decision to seek medical care by a previous study; women with serious complications were more likely than their counterparts to seek care from a health facility [44]. In Indonesia, it has been reported that women who report not having any complications during pregnancy had increased odds of underutilizing ANC services [45]. Nevertheless, women’s perception of pregnancy risk may differ from the medical definition in some significant aspects. According to national data, only about half of women in Yemen are informed about danger signs during pregnancy [26]. Consequently, knowledge about danger signs and symptoms of common health problems in pregnancy among women in Yemen was very poor [43]. Therefore, more awareness is needed to increase women’s knowledge and understanding of the benefits of early and continuous utilization of ANC services during pregnancy. More important is that the health system-related barriers to the use of ANC and PNC services need to be addressed.

In summary, our study identified important factors which act as barriers or facilities to the use of maternal health services by rural women. Despite these important findings, we acknowledge that our research team had limited choices in selecting the study areas and participants, which may have influenced our results. In addition, we used a non-probability sampling technique which limits the generalization of our findings. For instance, women who visited the maternity and childhood health centres were recruited for this study. It can be assumed their perspective of the public health system may differ significantly from women who have not used the public health system for prenatal or delivery care. However, the qualitative approach allowed for the exploration of the barriers to the provision and use of health services from the participants’ perspective. The use of triangulation also enabled us to confirm the responses across the various participant groups.

## Conclusions

Our analysis identified many factors, including factors relating to policy, health providers, and patients, which act as facilitators or barriers to the use of maternal health services in two rural areas of Yemen. More importantly, these factors, work independently or jointly to influence the use of ANC and postnatal care services among rural women. The public health system in the study area was characterized by staff inadequacies, lack of essential medical equipment, and inadequate drugs. As a result, the provision of maternal health services, as well as health promotion activities on reproductive health, were limited. On the hand, poor quality of health services, costs of services, and perceived need for care during pregnancy and after childbirth were perceived barriers to the utilization of maternal health services by women. These barriers need to be addressed to improve the use of reproductive health services by women in Abyan and Lahj of Yemen.

## Supplementary information

**Additional file 1.**

## Data Availability

The datasets used and/or analysed during the current study available from the corresponding author on reasonable request.

## Abbreviations

ANC: Antenatal Care
C4D: Communication for development
DHS: Demographic Health Survey
FGDs: Focused Group Discussions
IDIs: In-Depth Interviews
LMICs: Low and Middle-Income Countries
MoPHP: Ministry of Health and Population
PNC: Postnatal Care
WHO: World Health Organizations
