# Supplementary material for: Health information and health-seeking behaviour in Yemen: perspectives of health leaders, midwives and mothers in two rural areas of Yemen
Source: BMC Pregnancy Childbirth. 2020 Jul 14;20:404. doi: 10.1186/s12884-020-03101-9 (PMC7359610; doi:10.1186/s12884-020-03101-9)
Supplement: Supplementary file 1 — Additional file 1. [file 12884_2020_3101_MOESM1_ESM.docx]

**In-Depth Interview guide for health leaders**

| - Where do rural mother receive health information on maternal health services?  - Is there any difference in maternal health care education programmes between urban and rural areas? How are these programmes provided? (Probe)  - In your opinion, how can you evaluate the current maternal healthcare education programmes which are provided to rural mothers? (Probe)  - What are the challenges you face in the transfer of health information to all mothers? How could in order to overcome such these obstacles? (Probe)  - What are the main factors influencing mothers' decision to seek health care services from public health facilities?  - How can the current health information programmes help to improve mothers’ and their children’ health status? (Probe)  - Is there any cooperation between the ministry of health and the Non-governmental organizations (NOGs) and the international health organizations to improve maternal healthcare education process to reach all rural mothers? (Probe)  - Do you have anything about the discussion that you would want to share with me? |
| --- |

**In-Depth Interview guide for midwives**

| - How would you describe the current health education and promotion programmes on maternal health in your facility? - How would you describe the healthcare services that are provided to rural mothers at the reproductive healthcare centres? (Probe) - In your opinion, how could you help to transfer health messages to all mothers? Do you think you are qualified enough to give mothers the right health advice? If No: why? (Probe) - What are the challenges you face in effectively carrying our health education and promotion activities on maternal health? (Probe) - In your opinion, what factors influence the use of antenatal and postnatal health care from public health facilities by rural mothers? (Probe) |
| --- |

**Focus group discussion guide for mothers**

| - What do you understand by antenatal care? - In your opinion, who needs antenatal health care services and why? - When is the right time to start antenatal care during pregnancy? - If you are pregnant, where do you go for antenatal care? How many times did you attend before delivery?(Probe) - Could you describe your experiences of antenatal care? - What is the main purpose of visiting the reproductive healthcare centres during pregnancy and after delivery? What challenges do you face in receiving maternal health services from the reproductive healthcare centres? (Probe) - Where else do you go to receive maternal health services during pregnancy? Why do you go there? - Where did you deliver your last baby? What were your reasons for delivering there? (Probe) - Have you received any health information? What are your health information sources? What were the health information topics? Do you think you have enough health information to protect yourself and your babies from complications or diseases? (Probe) - Are you satisfied with the health information and the health services that are provided to you during pregnancy and after delivery? Why? (Probe) |
| --- |
